# Editorial: From Biomass to Advanced Bio-Based Chemicals & Materials: A Multidisciplinary Perspective

**DOI:** 10.3389/fchem.2020.00131

**Published:** 2020-02-25

**Authors:** Florent Allais, Xavier Coqueret, Thomas Farmer, Warwick Raverty, Caroline Rémond, Gabriel Paës

**Affiliations:** ^1^URD Agro-Biotechnologies Industrielles (ABI), CEBB, AgroParisTech, Pomacle, France; ^2^Institut de Chimie Moléculaire de Reims, CNRS UMR 7312, Université de Reims Champagne-Ardenne, Reims, France; ^3^Department of Chemistry, University of York, York, United Kingdom; ^4^Department of Chemical Engineering, Monash University, Melbourne, VIC, Australia; ^5^FARE Laboratory, INRAE, Université de Reims Champagne-Ardenne, Reims, France

**Keywords:** biomass, lignocellulose, cellulose, hemicellulose, lignin, biochemicals, biomaterials, catalysis

Lignocellulosic biomass, one of the major feedstocks of the emerging bioeconomy, will play a key role in the replacement of petroleum-based chemicals and materials and will help to fight against global warming by providing renewable, carbon-neutral sources of energy. Nevertheless, because of its chemical and structural complexity, the transformation of lignocellulose into commodity and higher-valued products requires a combination of physical, biological, and chemical processes and a better understanding of both its composition and architecture at different scales, in order to render this transformation efficient and economically competitive. Importantly, lignocellulose transformation can also bring to the market novel and sustainable chemicals that can lead to new applications and new industries that can replace the mining and burning of fossil carbon. In particular, exploitation of aromatic molecules in lignin and of cellulose and hemicelluloses can produce biobased solvents, surfactants, plasticizers, functional additives for nutrition and cosmetics and life-saving medicines. In addition to this broad range of chemicals, cellulosic fibers, and particles fractionated from lignocellulosic biomass are increasingly used to produce composite materials. Overall, this Research Topic aims to illustrate how complementary approaches are relevant in addressing questions regarding the deconstruction of different forms of lignocellulosic biomass and the various processes required to turn them into valuable bio-based, renewable products.

The Research Topic comprises a collection of 16 original contributions: 14 research papers, one review and one mini-review dedicated to the modification, characterization and preparation of bio-based chemicals and materials using advanced chemical, physical and biochemical routes.

The review by Glasser is dedicated to lignin applications in materials, presenting how this group of aromatic bio-polymers can readily be tailored to get specific properties through chemical modification, and how compatibilization strategies such as lignin chemical functionalization can overcome the usual limitations encountered with unmodified-lignins for making advanced materials. The mini review by Zoghlami and Paës presents an up-to-date survey of the relative impact of chemical and structural factors on lignocellulosic biomass recalcitrance and of the most advanced techniques to evaluate these factors, together with recent spectral and water-related measurements to predict ease of hydrolysis.

Besides these two review articles, several articles detail how pre-treatments are necessary to facilitate subsequent reactions in biomass processing. Sipponen and Österberg assess aqueous ammonia for the separation of lignin from hydrothermally pre-treated wheat straw prior to enzymatic saccharification. The resulting lignin particles have potential in water remediation applications. A contribution by Lahtinen et al. focuses on extraction by pressurized hot water treatment, characterization, and concentration of phenolic residues from wood and their role in physical and oxidative stabilization of emulsions. Araya-Farias et al. have implemented a statistical approach to optimize several parameters during the pre-treatment of biomass with ionic liquids, leading to a useful mathematical model that predicts sugar release after enzymatic hydrolysis.

Chemical routes provide new perspectives for upgrading biomass to bio-based products. Flourat et al. have designed a new chemo-enzymatic route to synthesize advanced lignin models. Starting with lignocellulose-derived ferulic acid the first syntheses of two trimers of monolignol G possessing side-chains and both robust β-5 bonds and labile β-*O*-4 bonds have been achieved. Moore et al. report another chemo-enzymatic pathway, demonstrating that iodine supported on acidic alumina is a very effective catalyst for aza-Michael additions on bio-based itaconate polyesters. This reaction might reduce lengthy reaction sequences and produce new bio-based polyesters. The action of pyranose dehydrogenases on various xylo-oligosaccharides was assayed by Karppi et al. and demonstrated the potential to synthesize bifunctional molecules directly from hemicellulose fragments. This discovery could be the starting point for further derivatization and/or polymerization into bio-based chemicals and materials. Kammoun et al. investigated the possibility of using seawater to convert C5- and C6-sugars by hydrothermal conversion to furanic and acidic products, especially to lactic acid, offering interesting new chemical transformations in combination with dehydration catalysts or in biphasic systems.

Finally, the last group of articles illustrate some useful conversions of biomass into bio-based materials. In the case of lignin, Szabó et al. performed immobilization of lignin on carbon fiber and highlighted the importance of considering both the physicochemical properties of the matrix and of the polymer in order to design bio-composites with well-defined properties. Reactive extrusion was used by Milotskyi et al. to esterify kraft lignin with succinic and maleic anhydrides. Extensive characterization of the resulting materials demonstrated their potential for further polymerization or copolymerization and for combination with other polymers to produce new plastics with a significant bio-based content. Successful esterification of several industrial lignins with maleic acid in an acidic ionic liquid was achieved by Husson et al. resulting in increased solubility in polar and protic solvents without affecting thermal stability and opening the way to new polyolefin-lignin blends. Using vanillin as substrate, Savonnet et al. have synthesized bio-based aromatic diamines which were tested as curing agents for the design of bio-based epoxy thermosets. Further optimization of the synthetic pathways described are still necessary but thermomechanical properties in terms of glass transition temperature and char residue are considered promising.

Other bio-based polymers have demonstrated potential to create completely novel materials. For instance, a double-reagent simultaneous functionalization was used by Wang et al. in order to prepare porous polysilsesquioxanes having bifunctional groups. These novel materials were found to be nanoparticulate with morphology that could be controlled with the proportion of the reactants. Their meso- or macro-porous features are considered to have potential applications in environmental remediation. Moyer et al. showed that nanocellulose can serve as an effective templating agent by introducing controlled porosity and morphology to enhance surface area and introduce higher order architecture within catalyst particles, resulting in improved catalytic activities. Le Guen et al. have prepared and characterized biomass powders compounded in polylactic acid by twin screw extrusion to produce filaments for fused-deposition modeling 3D printing. The mechanical properties of the printed samples were found to be more affected by the printing direction than by the presence or type of biomass powder used.

The Editors hope that this collection, showing a great diversity of transformation pathways, characterization techniques and practical applications, will be of significant interest to Frontiers readers and will inspire significant progress in the field of biomass valorisation.

## Author Contributions

All authors listed have made a substantial, direct and intellectual contribution to the work, and approved it for publication.

### Conflict of Interest

The authors declare that the research was conducted in the absence of any commercial or financial relationships that could be construed as a potential conflict of interest.

